# Supplementary material for: Macrophage Migration Inhibitory Factor Suppresses Natural Killer Cell Response and Promotes Hypoimmunogenic Stem Cell Engraftment Following Spinal Cord Injury
Source: bioRxiv. 2025 May 7:2025.05.06.652516. Preprint. [Version 1] doi: 10.1101/2025.05.06.652516 (PMC12247959; doi:10.1101/2025.05.06.652516)
Supplement: 1 [file NIHPP2025.05.06.652516v1-supplement-1.pdf]

bioRxiv preprint doi: https://doi.org/10.1101/2025.05.06.652516; this version posted May 7, 2025. The copyright holder for this preprint (which was not certified by peer review) is the author/funder, who has granted bioRxiv a license to display the preprint in perpetuity. It is made available under aCC-BY 4.0 International license.

Supplementary Materials

Supplementary Table 1. Guide RNA sequences for CRISPR mediated B2M and CIITA knockout

Gene Name	B2M	CIITA
Transcript ID	ENST648006.3	ENST00000324288.12
guide RNA 1	GGCCGAGAUGUCUCGCUCCG	CACAGCUGAGCCCCCCACUG
guide RNA 2	ACUCACGCUGGAUAGCCUCC	GGCUCCUGGUUGAACAGCGC
guide RNA 3	CGGAGCGAGAGAGCACAGCG	CCCCUAACAUACUGGGAAUC
Forward primer	ACAGCAAACTCACCCAGTCTAG	TGAGAGCTTGGGGTCCCTTA
Reverse primer	CCAGTCTAAGGGAAGCAGAGC	CTGAGGCATGTTCTCTGCCA
Sequencing primer	AAACTCACCCAGTCTAGTGC	GGTAGGGGCTTGGAGCTAAC

Supplementary Table 2. Primer sequences for qPCR

Gene Name	Forward	Reverse
GAPDH	AATCCCATCACCATCTTCCAG	AAATGAGCCCCAGCCTTC
MIF	GTTCATCGTAAACACCAACGTG	GAAGGCCATGAGCTGGTC
